# Expanding Opportunities for Science, Technology, Engineering and Mathematics Subjects Teaching and Learning: Connecting through Comics

**DOI:** 10.21315/mjms2019.26.4.15

**Published:** 2019-08-29

**Authors:** Mazlini Adnan, Jafri Malin Abdullah, Laili Farhana Md Ibharim, Tan Wee Hoe, Dahlia Janan, Norazilawati Abdullah, Noorzeliana Idris, Amila Saliza Abdul Wahab, Ahmad Nizam Othman, Mohd Ekram Alhafis Hashim, Nooraishah Md Said, Rosni Adnan, Sutinah Yahaya, Norziah Amin, Mohd Abaidi Mohd Noh, Nadira Idari Sufa’at, Rohana Abdullah, Yusmarliza Yusof, Zaiton Ahmad @Mat Reduaan, Normiahni Ahdary, Thangeswary a/p Annamallai, Fauziah Ali, Kanimohli a/p Kannasamy, Ridzuan Shukri, Thanalachamy a/p Simadiri, Tan Sai Wah, Nur Farah Atirah Baharudin

**Affiliations:** 1 Department of Mathematics, Faculty of Science and Mathematics, Universiti Pendidikan Sultan Idris, Perak, Malaysia; 2 Department of Neurosciences, School of Medical Sciences, Universiti Sains Malaysia, Kubang Kerian, Kelantan, Malaysia; 3 Brain and Behaviour Cluster, School of Medical Sciences, Universiti Sains Malaysia, Kubang Kerian, Kelantan, Malaysia; 4 Kementerian Pendidikan Malaysia, Putrajaya, Malaysia

**Keywords:** comic STEM, Year One children, STEM education, mathematics, science, cognitive neurosciences

## Abstract

This study presents the results of a year-long project focused on analysis and reflection on working with comics by Year One students in Hulu Langat districts. This study presents the use of science, technology, engineering and mathematics (STEM) comics to help children understand certain physical phenomena and try to make students interested in mathematics and science subject. Thirteen excellent teachers of science and mathematics from the Hulu Langat district were involved in the analysis of syllabus Year One science and mathematics subjects and the preparation of scripts while the STEM comic illustrator was created by two lecturers from the Faculty of Art, Computing and Creative Industry from Universiti Pendidikan Sultan Idris, Perak Malaysia. The study is based on observations of changing perception of phenomena by children as a result of the use of comics. As a result, a STEM comic that contains ten series for Year One science and mathematics subjects has been successfully developed. This comic is expected to attract and enhance the achievement of Year One students in science and mathematics. Implication of this study, STEM comics can be used by teachers as science and mathematics teaching aids. Comics are proven to be a modern pedagogical strategy, which is starting to gain its popularity in teaching about mathematics and science. Comics can be very helpful tools in making science and mathematics concepts interesting, fun learning and comprehensible for a Year One children.

## Introduction

The word ‘comic’ comes from the Greek word ‘kōmōidía’ or ‘comedy’. Comics use pictures with words that are often combined with humour and can be found in a variety of contexts, such as newspapers. Though similar types of artistic approaches could include cartoons which usually involve drafts or animation, or graphic novels and a much more detailed story of episode.

The research focus on using comics as a pedagogical tool for teaching and learning dates back to the 1940s and mostly focused on emerging literacy (1, 2). Incorporating texts with visual representations while teaching young children to read contributed to increasing children’s attention due to novelty and incongruity, more elaborate retrieval strategies and positive emotions are associated with learning (1, 4, 5). McVicker (4) stated that, cartoons can be used to enhance and support the learning that goes on in any classroom in versatile and creative ways.

Although there are few experimental research designs in education, (6) and (7) had documented positive student learning gains when humour is used effectively in the course of instruction. Nagata (8) found that using *manga* (Japanese comics and cartoons) helped students in biochemistry ‘use additional information and provide cognitive-psychological and pedagogical-technical effects: They give students clues to remember what they have learnt and make biochemistry lectures exciting’ (p. 203). Martin (5) stated that “humour serves as a sort of mnemonic technique or memory aid, causing greater elaboration of information and therefore enhancing its transfer and storage in long-term memory” (p. 104). Through humour and pictures, comics can illustrate key points and ‘lighten’ the classroom setting. This type of dual processing, emotional (humour) and visual (pictures/text), can help level the playing field for students trying to accommodate abstract content. Martin (5) noted that humour should be directly related to the content and student learning, not just merely for entertainment. Selectivity of the stimuli is important with attention devoted to novelty, incongruity and surprise, while refraining from sarcasm and crassness (9).

## STEM Comics

When students are faced with learning abstract contents, creating meaningful teaching and learning opportunities is a challenge for many educators. Thus, STEM comics have been developed as one of the steps to produce meaningful teaching aids. STEM comics is a special comic for Year One students integrating science and mathematics into one comic. This comic is derived from a study which grants given by the Educational Planning and Research Division (EPRD) division. The title of the study was ‘STEM comics construction and testing on the achievement of science, mathematics and higher order thinking skills (HOTS) among Year One school students in Hulu Langat district, Selangor’.

**Figure 1:**
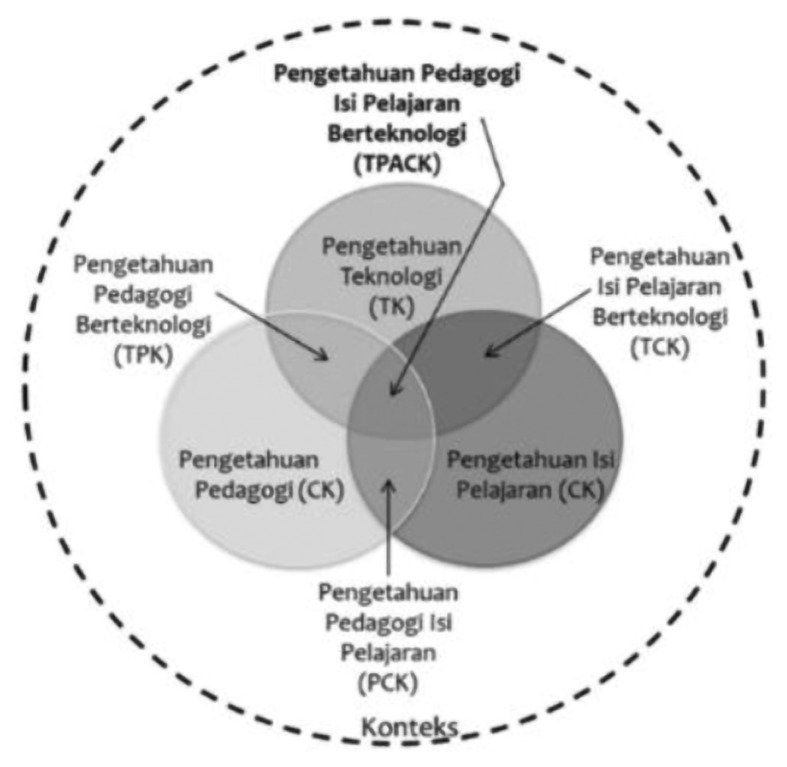
Model of knowledge technology, pedagogy and content (10)

The STEM comics construction phase uses the TPACK model as shown in Figure 1 beginning with the preparation of knowledge and pedagogical content involving thirteen outstanding Hulu Langat science and mathematics teachers. Four topics for science subjects and five topics for second-term mathematics are selected from the Curriculum and Assessment Standard Document (DSKP), Primary School Standard Curriculum (KSSR) as shown in Table 1.

The constructive alignment of the content of knowledge and pedagogy between science and mathematics subjects is first implemented before the mapping of the comic storyline is done. In this comic, there are five main characters: Professor Megat Adam (neuro-gardener sifoo), Megat Amir (neuron man), Princess Hanna (neuron woman), Megat Alif (neuron boy) and Princess Sarah (neuron girl). From technological aspects, the STEM comics illustration is translated in the form of comics as a result of the storyline. Illustrators consisting of lecturers and UPSI students use Adobe Photoshop and Adobe Illustrator software. The STEM comics go through the process of revising the appropriate language and graphics aspect according to the Ministry of Education (MOE) standards.

There are ten series in comics include a series of introduction. Each has its own theme. The following themes are included for each series which are Introduction, Series 1–Playground, Series 2–Happy Birthday Sarah, Series 3–Month Committee of Science And Mathematics, Series 4–The Rain Oh Rain, Series 5–Sports, Series 6–Rotten, Series 7–Vegetable Project, Series 8–Going Back to Hometown, Series 9–Picnic in Port Dickson.

## Methodology

The STEM comics were conducted at eleven primary schools in the district of Hulu Langat namely Sekolah Kebangsaan Kajang, Sekolah Kebangsaan Kajang Utama, Sekolah Kebangsaan Sri Jerlok, Sekolah Kebangsaan Semenyih, Sekolah Kebangsaan Kg Rinching, Sekolah Kebangsaan Bandar Seri Putera, Sekolah Kebangsaan Bangi, Sekolah Kebangsaan Tasek Permai, Sekolah Kebangsaan Pandan Jaya, Sekolah Kebangsaan Tun Hussien Onn 2 and Sekolah Kebangsaan Batu 9. A total of 307 students were selected from a class that did not conduct teaching and learning in dual programmes. Additionally, selected students also have no learning and reading problems. The selection of the sample was using a randomised stratified method of two schools selected from each zone in the district of Hulu Langat.

The comic sessions by science and mathematics teachers were carried out for a week and the comic test was implemented after that period. Comic test sessions are conducted in the classroom with the help of teachers and researchers using the Single-Group Post Test Only Quasi-Experiment method. Instruments used during the test session were Inventory Measurement of Interest in Science and Mathematics, Usability Measurement Inventory, Science Achievement Assessment as well as Mathematical Achievement Assessment. After data is collected, the data is analysed using descriptive statistics (mod and median) to determine the interests, usability and achievement of students’ science and mathematics after using STEM comics.

## Results and Discussion

Comics can be used to establish a positive affective context and interest in the theories of teaching and learning, and encourage students to continue to reflect and think critically on the best practices for learning and engagement. A heightened sense of enjoyment in the class and the appreciation for teachers who use humours in the classroom have been found in experimental studies (10).

### Interest in Comics, Science and Mathematics

The result shows that an interest in comics was high with a mean value of 2.63, while interest in science and mathematics, respectively, is 2.65 and 2.63. This shows that, as a whole, most of Year One pupils are interested in comics reading. Additionally, they also agree that STEM comics can attract students to learn science and mathematics.

### Usability of Comics

The usability value of comics is based on five sub-constructs that are controls, reader satisfaction, accessibility, reader efficiency and learning. It is found that four out of five sub-constructs show a high mean value of readership satisfaction (2.70), accessibility (2.53), readership efficiency (2.52) and learning (2.74). Overall, the Year One students agreed that STEM comics could be used in science and mathematics as teaching aids.

## Conclusion

This paper highlights on how to use comics to illustrate educational psychological concepts and promote student learning. Because humourous illustrations and situations are often associated with positive emotions, educators have acknowledged the beneficial effects of such devices (5). Using alternative methods, such as humours or comics to guide students in constructing knowledge, creates a richer understanding of concepts that can be applied into actual learning settings. It can also enhance attention and help with retrieval strategies. By encouraging innovative methods in teaching, educators can move theory from an intellectual exercise to a pedagogical tool for critical thinking and instructional decision making.

The findings of the study shows that the comics developed are objective and can be implemented well. In addition, it is found that all series of comics produced can be implemented and appropriate to the target group. This means that all the series and themes found in the resulting STEM comics are at a good and satisfactory level. Next, they also agree that STEM comics can attract students to learn science and mathematics. Based on the findings of the achievement test, STEM comics can help students in improving the achievement of science and mathematics. It is hoped that this STEM comics can be used as a meaningful material for Year One students so that it can help increase students’ interest and achievement in science and mathematics.

## Figures and Tables

**Figure 2:**
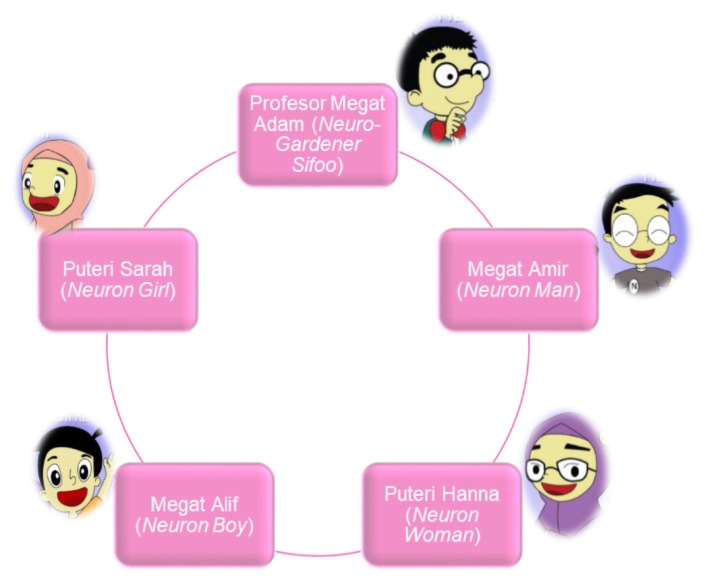
Main characters of STEM comics

**Figure 3:**
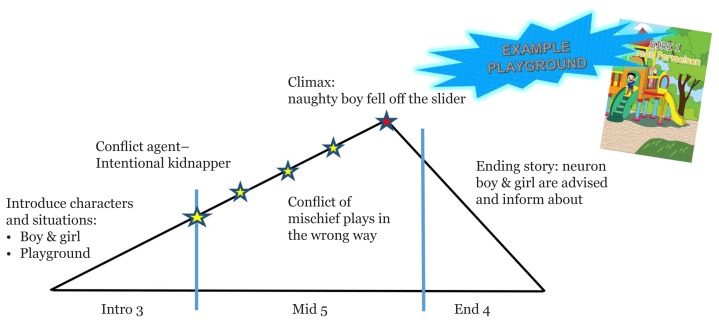
Log line: neuron boy and girl–playground–conflicts of naughty boy misuse the game tools

**Figure 4:**
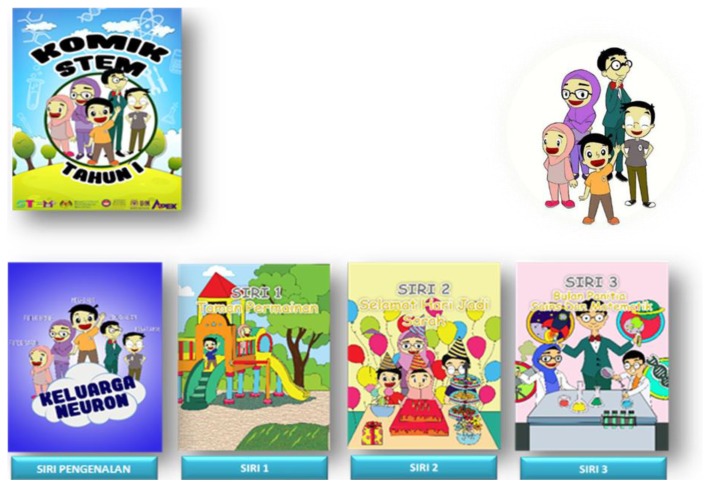
Front cover and series of STEM comics

**Figure 5:**
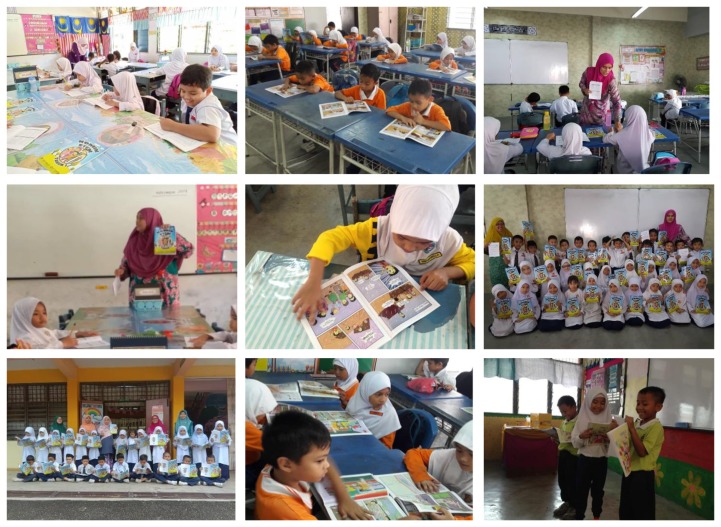
The implementaion of STEM comics during teaching and learning session

**Table 1:** Topics in science and mathematics and series matched

Science	Series	Mathematic	Series
Construction of base shape	Series 1, 2 and 3	Space	Series 1
Absorption	Series 4, 5 and 6	Day and month, face of the clock	Series 2
Land	Series 7 and 8	Data management	Series 3
The face of the earth	Series 9	Size measure	Series 4, 5, 6, 7
		Two-dimensional	Series 9
